# Biochemical liver damage during gender affirming therapy in trans adults assigned female at birth: a meta-analysis

**DOI:** 10.1007/s40618-024-02418-y

**Published:** 2024-06-22

**Authors:** D. Tienforti, G. Savignano, L. Spagnolo, F. Di Giulio, M. G. Baroni, A. Barbonetti

**Affiliations:** Andrology Unit, Department of Clinical Medicine, Life, Health and Environmental Sciences, University of L’Aquila, 67100 L’Aquila, Italy

**Keywords:** Gender-affirming hormone therapy, Gender incongruence, Testosterone, Liver, Hepatic injury

## Abstract

**Purpose:**

To assess the effects of testosterone (T)-based gender affirming hormone therapy (GAHT) on liver blood tests (LBTs) in assigned female at birth adults, using a meta-analytic approach.

**Methods:**

Prospective and retrospective studies were selected that reported the prevalence of biochemical liver damage (BLD) and LBTs changes during T therapy. Data collected included pre-and-during therapy alanine-aminotransferase (ALT), aspartate-aminotransferase (AST), gamma-glutamyl-transferase (GGT), and alkaline phosphatase (ALP) mean concentration values.

**Results:**

The prevalence of BLD in 14 studies on 1698 subjects was 1% (95% CI 0.00–3.00; I^2^ = 14.1%; p = 0.82). In 17 studies on 2758 subjects, GAHT was associated with a statistically (but not clinically) significant increase in AST, GGT and ALP at 12 months and ALT at 3–7 (MD: 1.19 IU/l; 95% CI 0.31, 2.08; I^2^: 0%), at 12 (MD: 2.31 IU/l; 95% CI 1.41, 3.21; I^2^: 29%), but with no more significant increase at 24 months (MD: 1.71 IU/l; 95% CI −0.02, 3.44; I^2^: 0%).

**Conclusions:**

Analysis of aggregate estimates confirms a low risk of BLD and abnormalities in LBTs, transient in most cases, during T-based GAHT, thus suggesting a limited need for careful liver monitoring in AFAB people.

## Introduction

The number of people seeking medical interventions for gender incongruence (GI) has increased worldwide over the last two decades and, with it, the demand for access to gender-affirming hormone treatment (GAHT)[1]. Since the latter usually involves the lifelong administration of drugs to commonly young individuals, the side effects need to be not minimized.

The liver, expressing receptors for androgens [2] and providing metabolism of these hormones [3], is sensitive to the effect of both endogenous and exogenous testosterone (T) [3, 4]. The correlation between hepatotoxicity and androgens has been attributed precisely to the role of the liver as the primary site of their clearance: synthetic steroids are, in fact, frequently modified to prevent first-pass hepatic metabolism, which, by preventing their elimination, exposes the liver to the risk of toxicity [5]. In particular, the chemical substitution responsible is, above all, 17-α alkylation, which, in addition to allowing oral administration, renders androgens resistant to immediate degradation; 17-β esterification, the other main modification in the formulation of synthetic steroids, requires parenteral administration, results in greater potency and duration of action, and is less associated with liver damage than the former [6, 7]. However, because oral 17-alkylated T is currently not recommended as a therapeutic approach, severe liver toxicity described in the past [8, 9] is not expected with parenteral or transdermal T used in both cis- and transgender patients. Past concerns reported in earlier editions of guidelines on GAHT [10, 11] regarding T liver toxicity have been alleviated by more recent reports [12–14] indicating the risk of serious liver disease is minimal [15].

In the majority of the studies on assigned female at birth (AFAB) transgender population, T-based GAHT induces, as a rule, a slight increase in the level of ALT and AST [16] the clinical significance of which is, in general, minimal [17]. However, the possibility of liver damage caused by long-term administration of T esters during GAHT remains an open question [16].

In light of the above, the present study aims to verify and quantify, using a meta-analytic approach, the effects on the liver of T-based GAHT in AFAB subjects.

## Methods

The study was conducted according to the statement preferred reporting items for systematic reviews and meta-analyses protocols (PRISMA-P) [18]; it also complies with the guidelines for meta-analyses and systematic reviews of observational studies (MOOSE) [19]. Being a systematic review with meta-analysis, the study did not directly enroll human participants. The Declaration of Helsinki was adequately addressed, and no specific permissions were required for corresponding locations. The study protocol was registered in the international prospective registry for systematic reviews (PROSPERO) with registration number CRD42023486643.

### Systematic search strategy

A systematic search was carried out in PubMed, Scopus, Web of Science and Cochrane Library in order to identify the totality of studies published in English on this topic up to November 2023. The databases were queried by means of a purpose-built search string using the biomedical vocabulary Medical Subject Headings (MeSH) of PubMed. For the extraction of publications (records), the following terms were used: “transgender”, “AFAB”, “FtM”, “female to male”, “transmen”, “trans men”, “GAHT”, “gender-affirming hormone therapy”, “testosterone”, “androgen”, “liver”, “hepato*”, “GPT”, “glutamic pyruvic transaminase”, “GOT”, “glutamic oxaloacetic transaminase”, “ALT”, “alanine transaminase”, “AST”, “aspartate transaminase”, “GGT”, “gamma-glutamyltransferase”, “ALP”, “alkaline phosphatase”, “acute liver injury” and “hepatotoxicity”. To combine these key terms we used the Boolean operators ‘AND/OR’. If it was not clear from reading the abstract whether the study contained relevant data, the full text was retrieved. Finally, in addition to the identification of eligible studies, we performed the detection of possible additional studies by means of a manual search in relation to the references cited in the articles as a whole.

### Inclusion and exclusion criteria

The selection of publications for inclusion was carried out in several stages. In the identification phase, querying the databases identified potentially eligible studies that could be included in the meta-analysis. Following the removal of repeated articles (same publication found in more than one database), in the second phase, studies of possible interest were screened by reading the title and abstract. In the third phase, the remaining articles were assessed in full-text for eligibility. Observational studies, both prospective and retrospective, as well as longitudinal intervention studies were considered eligible, while non-experimental descriptive studies, studies conducted on populations other than the one of interest, studies in which endpoints other than those being analyzed were evaluated, studies with an experimental design other than the one of interest and studies with incomplete or inaccurate data were excluded. Two independent reviewers (F.D.G., L.S.) assessed the full text of all selected studies to establish eligibility, and any disagreements were resolved through an open discussion involving a third reviewer (D.T.). The flow-chart proposed by Page et al. [20] was used to schematize the steps for the inclusion of studies.

### Quality assessment

The methodological quality of the included articles was established using the quality assessment tool for quantitative studies developed by the Effective Public Health Practice Project (EPHPP) [21]. This quality assessment tool, used for intervention studies as well as randomized controlled and case–control studies, was also validated for systematic reviews [22]. It considers the following domains: selection bias, study design, confounding factors, study blindness, data collection method and losses at follow-up. The quality of each domain can be indicated as strong, moderate or weak, and in the overall judgement the quality can be considered strong if no weak score was assigned, moderate if only a weak judgement was assigned to one of the domains and, finally, weak if two or more weak judgements were assigned to several domains.

### Data extraction

Data were extracted from the studies selected by two independent reviewers (D.T., G.S.). The primary outcome assessed was the prevalence of BLD, defined as a twofold or threefold increase -depending on the definition chosen by each considered paper- from the upper limit of normal for the assigned sex at birth (female) in ALT and/or AST values. The secondary outcome assessed was the mean pre/post-treatment difference in the ALT values, considered the most specific marker of liver damage being almost exclusively present in hepatocytes, analyzed at baseline and after 3–7, 12 and 24 months of therapy, and in the AST, GGT and ALP values, analyzed at baseline and at 12 months of therapy. Additional information extracted was the first author of the study, the year of publication, the country/geographical region of the study, the study design, the number, mean age and initial body mass index (BMI) of the participants, the type of T administered, the duration of follow-up in months and the blood parameters investigated in the study.

### Statistical analysis

The overall prevalence of BLD was estimated by means of DerSimonian and Laird’s random-effects model [23]. The 95% confidence intervals (CI) of the prevalence reported in the individual publications were estimated in relation to the proportion of BLD cases and the sample size using Clopper and Pearson’s binomial exact method. The effect of the therapy on LBTs was assessed using the mean difference (MD) with a 95% coefficient interval (CI) between post-treatment and baseline values with Mantel–Haenszel estimates. The Cochran’s χ^2^ (Cochran’s Q) and I^2^ tests were carried out to analyze statistical heterogeneity between the results of different studies: I^2^ > 50% and/or p < 0.05 indicated substantial heterogeneity [24]. Data were combined using a random effects model. Even when a low heterogeneity is detected, a random-effects model should be applied, because the validity of tests for heterogeneity can be limited with a small number of component studies. Publication bias was explored through the funnel plot [25] and Egger’s test [26]. Data were analyzed using the Review Manager of the Cochrane Library (version5.3; The Nordic Cochrane Centre, The Cochrane Collaboration, Copenhagen, Denmark) and the R statistical software (version 3.6.3, 2020; The R Foundation for Statistical Computing, Vienna, Austria) with the “metafor” package.

## Results

### Study selection

**Fig. 1:**
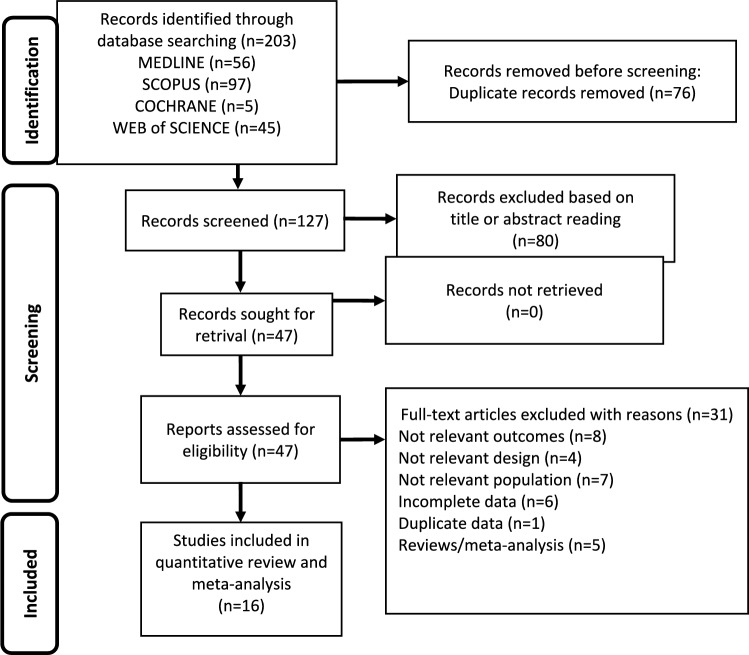
Flow diagram showing an overview of the study selection process

Searching from database yielded 203 studies and searching from other sources 11, for a total of 214. Removal of duplicates resulted in a total of 127 publications, of which 80 were judged to be irrelevant simply by reading the title and abstract. Thus, as shown in Fig. 1, 47 articles were identified, of which 16 met the inclusion criteria [1, 27–41]. Details of the studies included in the quantitative synthesis are summarized in Table 1.

**Table 1:** Characteristics of the included studies

Author	Year	Country	Study design	Sample size	Age (years)	BMI	Testosterone formulation	Follow-up (months)	Parameters	Cases of BLDs
Boekhout-Berends [22]	2023	Netherlands	Retrospective	1023	24.8 ± 8	25 ± 5.9	TG, iET, iTU	0–12	AST, ALT, GGT, ALP	NA
Chandra [23]	2010	USA	Prospective	12	29 ± 9	27.5 ± 18.2	iET, iTE, iTC	0–12	AST, ALT	0
Fernandez [24]	2016	USA	Retrospective	19	30 ± 7.6	28.1 ± 9.2	NS	0–(3–6)–(6–18)	AST, ALT	0
Gava (1) [25]	2018	Italy	Retrospective	25	29.8 ± 4.8	22.5 ± 2.6	iTU	0–12–24–36–48–60	AST, ALT	0
Gava (2) [25]	2018	Italy	Retrospective	25	30.4 ± 7.1	23.3 ± 4.8	iTE	0–12–24–36–48–60	AST, ALT	0
Gava [26]	2021	Italy	Prospective	7	35 ± 5.6	23.3 ± 3.5	iTU	0–12	AST, ALT	0
Jacobeit [27]	2009	European multicentric	Prospective	17	34 ± 7	28.3 ± 2.8	iTU	0–6–12–18–24–30–36	AST, ALT	0
Korpaisarn [28]	2021	Thailand	Retrospective	39	27.8 ± 6	23.6 ± 4.5	iTE	0–24	AST, ALT	0
Meriggiola [29]	2008	Multicentric	Prospective	5	34.4 ± 4.4	22.2 ± 2.1	iTU	0–12	AST, ALT	0
Meyer [1]	2020	Germany	Retrospective	233	27.3 ± 6.6	23.7 ± 4.9	TG, iTU	0–(3–4)–(10–14)–(36–48)	AST, ALT, GGT	5
Mueller [30]	2010	Germany	Prospective	45	30.4 ± 9.1	24.1 ± 4.5	iTU	0–12v24	AST, ALT, GGT	0
Pelusi (1) [31]	2014	Italy	Prospective	15	30.9 ± 4.9	22.3 ± 3.8	iET	0–7–12	AST, ALT	0
Pelusi (2) [31]	2014	Italy	Prospective	15	28.2 ± 4.3	22.1 ± 4.2	iTU	0–7–12	AST, ALT	0
Pelusi (3) [31]	2014	Italy	Prospective	15	29.4 ± 4.5	23.9 ± 4.4	TG	0–7–12	AST, ALT	0
Schönauer (1) [32]	2021	Italy	Prospective	15	27	24.2 ± 2.2	iTE	0–6–12–18–24	AST, ALT	NA
Schönauer (2) [32]	2021	Italy	Prospective	8	27	27.4 ± 5.5	iTE	0–6–12–18–24	AST, ALT	NA
Stangl [33]	2021	European multicentric	Prospective	1044	25, 6.7	25.5 ± 5.6	TG, iET, iTE, iTU	0–3–12	AST, ALT, ALP, GGT	6
Vita [34]	2018	Italy	Retrospective	11	25.1 ± 3.7	22.6 ± 3.6	iTU, iTE	0–24	AST, ALT, GGT, ALP	0
Vlot [35]	2019	Netherlands	Prospective	132	25.2 ± 2.3	24.9 ± 1.5	iTU, TG, iET	0–12	AST, ALT, ALP, GGT	0
Wierckx [36]	2014	European multicentric	Prospective	53	24.5 ± 6.8	24.8 ± 5.3	iTU	0–12	AST, ALT	1

Age and BMI are expressed as mean ± DS

*BLD* biochemical liver damage, *ALP* alkaline phosphatase, *BMI* body mass index, *iET* testosterone esters (injectable), *GGT* gamma glutamyl transferase, *NA* not available, *NS* not specified, *iTC* testosterone cypionate (injectable), *iTE* testosterone enanthate (injectable), *TG* testosterone gel, *iTU* testosterone undecanoate (injectable)

### Quality assessment

The quality assessment based on the EPHPP is given in Table 2. Overall, most studies (14 of 16) received a methodological quality rating of ‘‘moderate’’ [1, 27, 28, 30–32, 34–41] and 2 studies resulted ‘‘weak’’ [29, 33]. The items ‘‘confounders’’ and “data collection methods” received the highest rating among all the included studies; on the contrary, the item ‘‘blinding’’ was the most lacking, as in none of the studies the participants and the research staff who assessed outcomes were blind to the study conditions. Four studies received a ‘‘moderate’’ or ‘‘weak’’ methodological quality rating in the item ‘‘withdrawals and dropouts’’ because of the large difference in the number of participants between initial enrollment and the end of follow-up [1, 27, 29, 33].

**Table 2:** Quality assessment of the included studies

Study	Selection bias	Study design	Confounders	Blinding	Data collection methods	Withdrawals and drop-outs	Global rating
Boekhout-Berends 2023 [22]	Moderate	Moderate	Strong	Weak	Strong	Moderate	Moderate
Chandra 2010 [23]	Moderate	Moderate	Strong	Weak	Strong	Strong	Moderate
Fernandez 2016 [24]	Moderate	Moderate	Strong	Weak	Strong	Weak	Weak
Gava 2018 [25]	Moderate	Moderate	Strong	Weak	Strong	Strong	Moderate
Gava 2021 [26]	Moderate	Moderate	Strong	Weak	Strong	Strong	Moderate
Jacobeit 2009 [27]	Moderate	Moderate	Strong	Weak	Strong	Strong	Moderate
Korpaisarn 2021 [28]	Moderate	Moderate	Strong	Weak	Strong	Weak	Weak
Meriggiola 2008 [29]	Moderate	Moderate	Strong	Weak	Strong	Strong	Moderate
Meyer 2020 [1]	Moderate	Moderate	Strong	Weak	Strong	Moderate	Moderate
Mueller 2010 [30]	Moderate	Moderate	Strong	Weak	Strong	Strong	Moderate
Pelusi 2014 [31]	Moderate	Moderate	Strong	Weak	Strong	Strong	Moderate
Schönauer 2021[32]	Moderate	Moderate	Strong	Weak	Strong	Strong	Moderate
Stangl 2021 [33]	Moderate	Moderate	Strong	Weak	Strong	Strong	Moderate
Vita 2018 [34]	Moderate	Moderate	Strong	Weak	Strong	Strong	Moderate
Vlot 2019 [35]	Moderate	Moderate	Strong	Weak	Strong	Strong	Moderate
Wierckx 2014 [36]	Moderate	Moderate	Strong	Weak	Strong	Strong	Moderate

### Primary outcomes: prevalence of biochemical liver damage

**Fig. 2:**
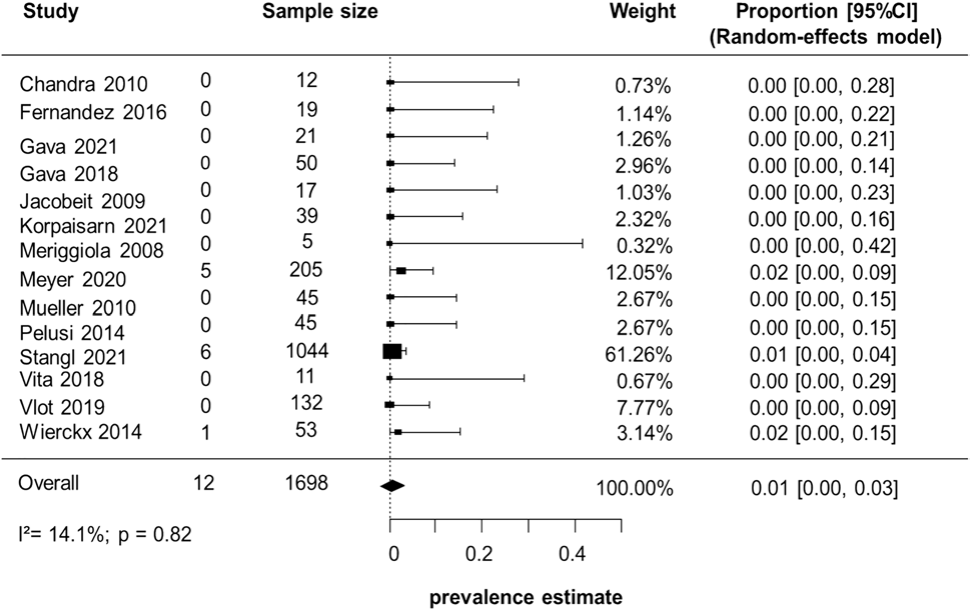
Biochemical liver damage prevalence estimate. Diamond indicates the overall estimate (width of the diamond represents 95% CI). Boxes indicate the weight of individual studies in the pooled result. *CI* confidence interval

Fourteen studies reported information on BLD in a total of 1698 AFAB subjects undergoing T-based GAHT: the overall prevalence (proportion) was 1% (95% CI 0.00–3.00), with minimal and non-significant heterogeneity (I^2^ = 14.1%; p = 0.82). In detail, 11 [28–36, 39, 40] of the 14 studies [28–36, 38–41], involving a total of 396 people, found no episodes of BLD, while in 3 studies [1, 38, 41], 6 out of 1044 [38], 5 out of 205 [1], and 1 out of 53 [41] subjects were reported (Fig. 2).

### Secondary outcomes

**Fig. 3:**
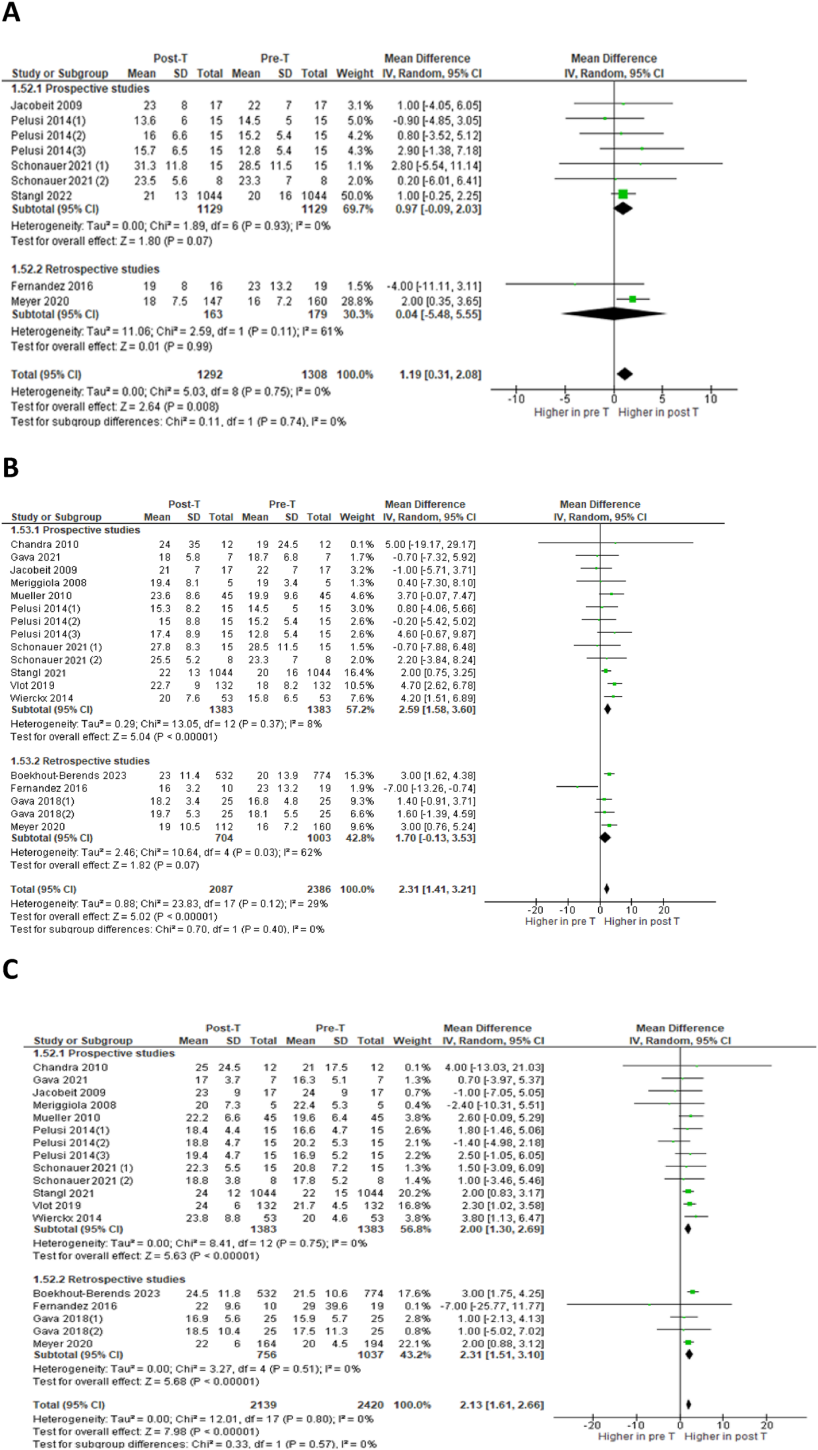

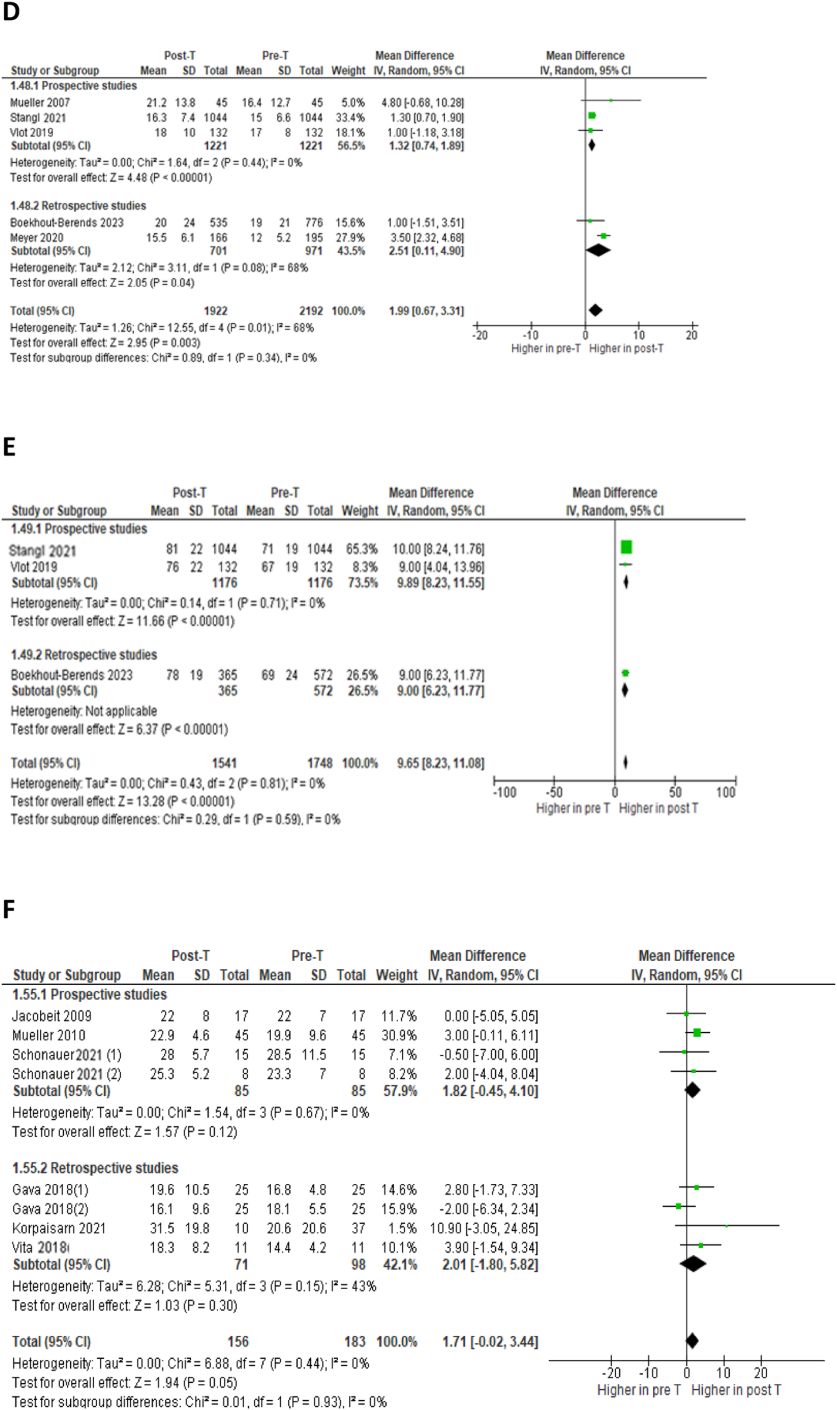
Forest plots of the effects of testosterone (T) therapy on ALT at 3–7 months **A**, ALT at 12 months **B**, AST at 12 months **C**, GGT at 12 months **D**, ALP at 12 months **E** and ALT at 24 months **F**. Diamonds indicate the overall effect estimates (and diamond width the 95% CI); squares indicate the weight of individual studies in the aggregate estimate. CI, confidence interval; df, degrees of freedom; IV, inverse variance

Six studies [1, 29, 32, 36–39] analyzed changes in ALT at 3–7 months of GAHT in a total of 1292 subjects (Fig. 3A): the difference between the aggregate means (MD) showed a statistically significant increase, in the absence of heterogeneity (MD = 1.19; 95% CI 0.31–2.08; p = 0.008; I^2^ = 0%, P_forheterogeneity_ = 0.75). At 12 months, fourteen studies [1, 27–32, 34–38, 40, 41] looked for changes in ALT and AST in a total of 2087 and 2139 persons, respectively (Fig. 3B and 3C): there was a statistically significant increase in both ALT (MD = 2.31; 95% CI 1.41–3.21; p < 0.00001; I^2^ = 29%, P_forheterogeneity_ = 0.12) and AST (MD = 2.13; 95% CI 1.61–2.66; p < 0.00001; I^2^ = 0%, P_forheterogeneity_ = 0.80), with no significant heterogeneity. At the same follow-up time (12 months), the meta-analysis of five [1, 27, 35, 38, 40] and three [27, 38, 40] studies, with a total of 1922 and 1541 subjects respectively, showed a statistically significant increase in levels of both GGT (MD = 1.99; 95% CI 0.67–3.31; p = 0.003; I^2^ = 68%, P_forheterogeneity_ = 0.01) and ALP (MD = 9.65; 95% CI 8.23–11.08; p < 0.00001; I^2^ = 0%, P_forheterogeneity_ = 0.81) (Fig. 3D and 3E). At 24 months, six studies [30, 32, 33, 35, 37, 39] analyzed the changes in ALT in a total of 156 persons (Fig. 3F): the difference between the aggregate averages was not statistically significant (MD = 1.71; 95% CI -0.02–3.44; p = 0.05; I^2^ = 0%, P_forheterogeneity_ = 0.44).

### Pubblication *bias*

**Fig. 4:**
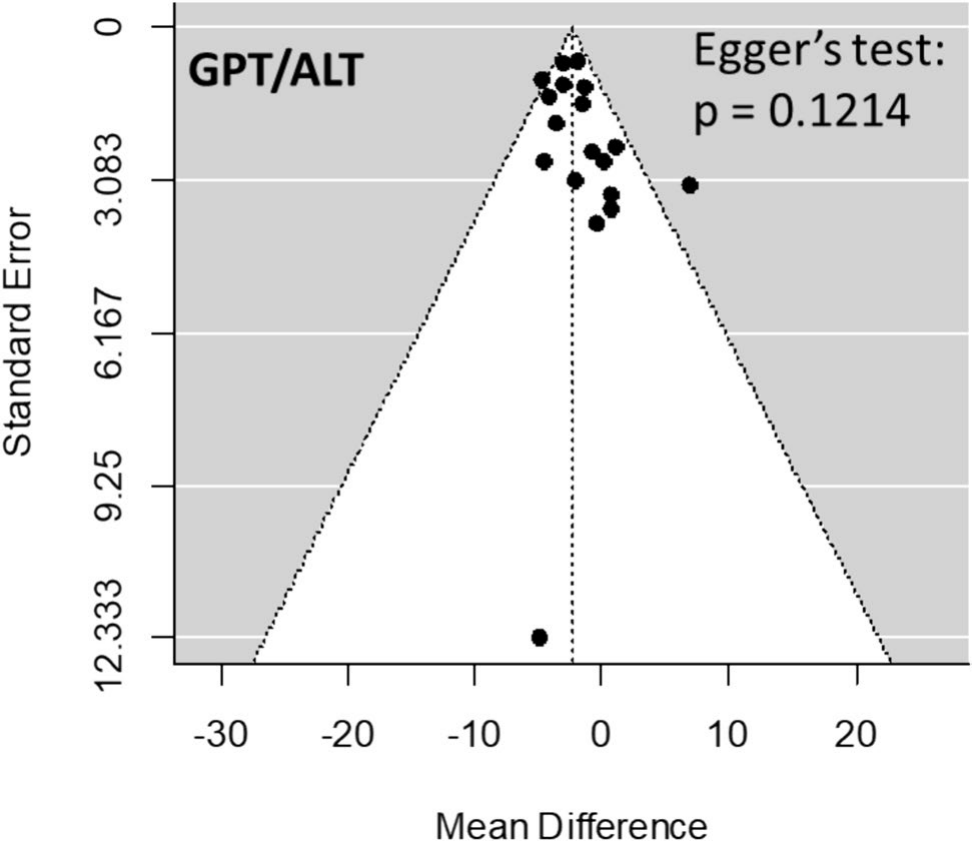
Assessment of publication bias

The relatively symmetrical shape of the funnel plot (Fig. 4) in relation to ALT concentration at 12 months of GAHT suggested the absence of publication bias, confirmed by the not significant p-value of Egger’s test (p = 0.1214).

## Discussion

While older T formulations using the orally 17-alkylated hormone were associated with hepatotoxicity, newer T formulations are thought to be safer. This notion is confirmed by the recent literature on T replacement therapy in cisgender hypogonadal men; whereas, there is less data on transgender populations.

The present study, which is, to our knowledge, the largest systematic literature review with meta-analysis on the effects on the liver of T-based GAHT in AFAB people, showed that T may change the serum concentration of liver enzymes to a statistically, albeit not clinically, significant extent.

Both T deficiency, which characterizes cisgender men with hypogonadism, and T excess, as in women with polycystic ovary syndrome, increase the level of liver blood tests in dysmetabolic pictures, including non-alcoholic fatty liver disease [3, 4, 42, 43]: this opposing correlation denotes a complex interpretation of the effects of T on liver cells.

The frequency and severity of side effects of chronic intake of androgens are considered to be dependent on various factors, including the formulation, route of administration, dosage and duration of use of the drugs as well as the response and sensitivity of individual subjects [6]. There are significant differences in adverse effects between the use of T and its synthetic derivatives under medical supervision and the uncontrolled, simultaneous use of multiple drugs at high doses for long periods of time [44] : individuals who abuse anabolic androgenic steroids [45, 46] for incongruous purposes, especially athletic and aesthetic purposes are those who mainly experience hepatic (cholestasis, pyelosis, neoplasia) and non-hepatic (hypertension, hypogonadism, infertility, aggression, mood disorders, kidney damage, addiction) sequelae [6, 7, 44] Therefore, the urgent need for studies on the hepatic effects of GAHT in AFAB persons cannot be ignored, as pointed out by Xu and colleagues [47], also because the scientific evidence to date is somewhat limited by the low number of participants and the short duration of follow-up [48].

In our study, the overall prevalence of BLD was 1%. In addition, there was an overall average increase in ALT and other markers of liver function between 3 and 12 months of administration. The change in the LBTs proved to be statistically significant but, probably, not clinically relevant considering the smallness of the increase. The figure for ALT, the most specific marker of liver damage, showed a trend towards stabilization at 24 months, supporting the idea that these changes were clinically not significant and transient in most cases.

The data of the present study were consistent with what has already been reported in the literature.

A retrospective study on morbidity and mortality, conducted in 293 AFAB people subjected to long-term administration of T esters or T undecanoate, found an increase in liver enzyme concentrations in 45 persons: in 13 of these, it was transient (< 6 months), in 20 it was persistent (≥ 6 months), in 12 it was associated with causes unrelated to hormone therapy and in none it was more than 2.5 times the upper limit of the reference values [49]. A longitudinal study, in 438 AFAB subjects, reported that T-based treatment resulted in a modest influence on the level of GPT/ALT and GOT/AST, which did not correlate with clinically significant changes in liver function [48]. Unfortunately, these studies were not included in the meta-analysis because of the lack of quantitative LBTs values [49] or undefined follow up duration [48]. As described by Dimakoupoulou et al. [50] in a review of eleven studies with a mean follow-up duration of 30 months and a mean age of participants of 24 years, T therapy is shown to produce no significant liver damage: ALT was not affected and AST remained in the reference range. A further systematic review of 6 studies [17] indicated that, in some of them, liver enzymes concentrations in AFAB persons undergone T did not change, while in others they increased, although not clinically relevant. The GAHT, therefore, would not cause negative effects on the liver [51] but only an adaptation of liver enzyme values to the reference ranges of the affirmed (male) gender [1, 27, 52] as is the case for numerous laboratory parameters, including red cell count, creatinine and lipid profile [52–55]. In fact, it has been described how T can increase the size not only of skeletal muscles but also of other organs [56, 57]: an increase in liver volume has been documented following T administration in healthy young men [58].

This meta-analysis has some limitations. First, the observational design of all included studies was without a control group; therefore, it is impossible to determine whether some of the observed effects are related to T therapy or to other factors. An important limitation, although limited to 12-month analyses, may be the dropout rate, which is sometimes substantial. In some cases, it was observed that the number of subjects completing the follow-up period was lower than the initially enrolled cohort. Calculating an average difference over a number of pre- and post-treatment observations not referring to the same people could be a source of bias if subjects lost to follow-up were randomly characterized by the same type of response to therapy. Finally, as shown in Table 1, most of the selected studies are from European countries, so special caution should be taken when extending these results to subjects from other geographical areas.

In the light of the data presented, it is therefore possible to consider liver damage a predominantly negligible risk in AFAB people on GAHT. In the studies considered in this meta-analysis, in fact, the changes in liver blood tests are slight, generally asymptomatic and reversible even without interruption of treatment, even if full recovery could require a prolonged time. To date, it is unclear whether the possible toxic effects of T-based therapy may be greater in people with preexisting liver disease or other related comorbidities (e.g. alcohol dependence, obesity, dyslipidemia, or insulin resistance associated with type 2 diabetes).

## Conclusion

The influence of T-based GAHT on BLT in the first 2 years appears modest and probably does not reflect clinically relevant changes in hepatic pathophysiology. The more specific marker of liver damage (ALT), after a slight initial increase, tends to stabilize its value as early as 24 months. These results confirm the substantial safety of GAHT for the liver, consistent with the routes of administration and dosages recommended by current clinical practice guidelines, in agreement with suggestions in the recent literature, which indicate that close monitoring of liver enzymes in the context of the risk of liver damage due to hormone therapy is not necessary for the transgender population [38]. Whether and to what extent this safety profile can be extended to individuals who are more vulnerable due to pre-existing liver disease remains to be clarified.

## Data Availability

Data sharing is not applicable to this article as no new data were created or analyzed in this study.
